# Clinical results and complications following surgical management of symptomatic os acromiale: a systematic review

**DOI:** 10.1186/s13018-018-1041-5

**Published:** 2019-01-23

**Authors:** Jennifer A. Purnell, Jonathan Bourget-Murray, Adam Kwapisz, Aaron J. Bois, Justin LeBlanc

**Affiliations:** 1 Department of Surgery, Section of Orthopaedic Surgery, University of Calgary, Calgary, Alberta Canada; 2 Clinic of Orthopaedics and Paediatric Orthopaedics, Medical University of Łódź, Łódź, Poland

**Keywords:** Os acromiale, Symptomatic, Surgical technique, Surgical outcomes, Complications

## Abstract

**Background:**

This review compares the outcomes and complication rates of three surgical strategies used for the management of symptomatic os acromiale. The purpose of this study was to help guide best practice recommendations.

**Methods:**

A systematic review of nine prospective studies, seven retrospective studies, and three case studies published across ten countries between 1993 and 2018 was performed. Adult patients (i.e., ≥ 18 years of age) with a symptomatic os acromiale that failed nonoperative management were included in this review. Surgical techniques utilized within the included studies include excision, acromioplasty, and open reduction and internal fixation (ORIF). The primary outcomes of interest included patient satisfaction. Range of motion and several standardized outcome measurement tools were also included in the final analysis.

**Results:**

Patient satisfaction was highest in the excision and ORIF groups, with 92% and 82% of patients reporting good to excellent postoperative results, respectively, compared to 63% in the acromioplasty group. All three patient groups experienced improvements in postoperative outcomes (i.e., active range of motion and patient-reported outcome scores). The excision group experienced a complication rate of 1%, while the acromioplasty group experienced a complication rate of 11% and the ORIF group a rate of 67%.

**Conclusion:**

This study reports on the largest sample of patients who underwent surgical treatment for a symptomatic os acromiale. We have demonstrated that excision of the os with meticulous repair of the deltoid resulted in the best clinical outcomes with the least complications. In healthy adult patients with a large os fragment and a normal rotator cuff, surgical fixation may provide increased preservation of deltoid function while offering good to excellent patient satisfaction. However, patients must be informed that a second procedure may be required to remove symptomatic hardware.

## Background

An os acromiale represents a failure of osseous union between the secondary ossification centers of the acromion (i.e., acromial apophysis) and is present in approximately 8% of the population [1, 2]. The majority of os acromiale cases are asymptomatic and are found incidentally; as a result, the true incidence of this shoulder problem is unknown. A small proportion of patients present with a painful shoulder that is attributed to inflammation at the pseudarthrosis, impingement of the rotator cuff, or arthritic changes of the acromioclavicular joint secondary to hypermobility of the unfused bony segment [2].

A number of surgical techniques have been described for the treatment of symptomatic os acromiale including fragment excision [3–10], open or arthroscopic acromioplasty [3, 6, 11], and open reduction and internal fixation (ORIF). A variety of fixation techniques have been reported with successful union and improvements in patient-reported outcome scores [12–21]. Currently, a universally accepted surgical technique to manage symptomatic os acromiale does not exist. In 2011, Harris et al. [22] systematically reviewed the radiographic and clinical outcomes of 115 patients (122 shoulders) that underwent surgical management of a symptomatic os acromiale. Since then, 9 additional studies (95 patients, 99 shoulders) have been published reporting the surgical results for symptomatic os acromiale. Despite such reports, a standard of care for the treatment of symptomatic os acromiale remains controversial.

At the present time, there is a paucity of high-level evidence supporting one surgical technique over another. The purpose of this systematic review was to compare the surgical outcomes and complications between three surgical techniques (i.e., fragment excision, acromioplasty, and ORIF) commonly used to manage symptomatic os acromiale. We hypothesize that patients will report comparable subjective outcomes following all surgical techniques. Furthermore, we anticipate an overall trend of improved objective outcome measures across all groups with the best results observed in the excision group. Additionally, we anticipate lower nonunion rates and higher complication rates when internal fixation is utilized.

## Methods

### Literature search

The present systematic review was performed in accordance with the Preferred Reporting Items for Systematic Reviews and Meta-Analyses (PRISMA) guidelines [23]. In January 2018, a comprehensive search for all level I–IV evidence published in the English literature using the online databases MEDLINE, PubMed, and Embase was performed. The purpose of this search was to identify eligible studies featuring postoperative patient-reported outcomes across patient groups managed by either surgical excision, acromioplasty, or surgical fixation (i.e., ORIF). The search terms “os acromial” and “os acromiale” were used to ensure all appropriate studies were captured. All relevant articles published up to and including June 2018 discussing the surgical management of symptomatic os acromiale in adult patients (i.e., ≥ 18 years of age) were identified. All prospective or retrospective studies, non-randomized comparison studies, and case series were considered for inclusion. If more than one study was conducted at the same institution, the article that had the most complete or recent data was selected. Studies were excluded if they met the following criteria: (1) basic science studies (e.g., biomechanical studies); (2) reviews, clinical guidelines/expert opinions, and technique articles without patient data; and (3) conference abstracts and gray literature.

### Study selection

**Fig. 1:**
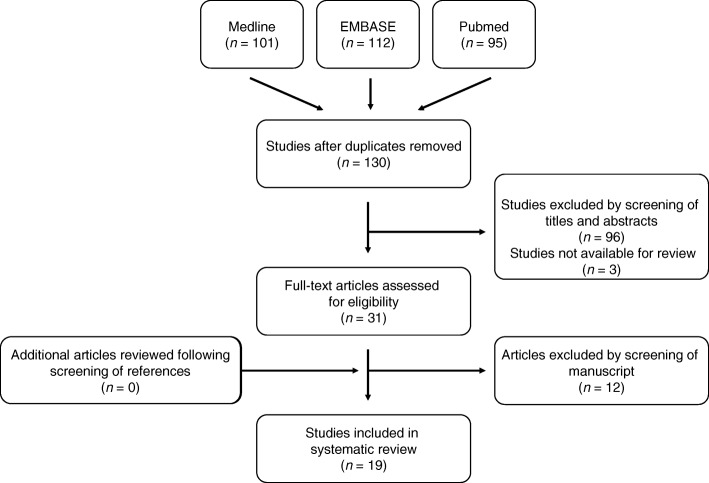
PRISMA flow diagram presenting the systematic review process used in this study

The search strategy identified 308 studies as outlined in Fig. 1. Duplicates were identified and removed from our reference manager, EndNote (Thomas Reuters, New York, NY). One hundred and thirty studies remained. All titles and abstracts were independently screened by two authors (JP and JBM) to determine study eligibility; there were no instances of disagreement between these two authors. This initial screen resulted in 31 studies that were subsequently retrieved (i.e., full-text manuscripts), independently reviewed, and accepted into the study if they met the inclusion criteria previously outlined. Of the 31 full-text manuscripts reviewed, 19 studies were included for the final analysis. The reference lists of all 19 studies were cross-referenced to capture additional studies missed by our initial search; no other studies were identified.

### Data extraction

Data extraction was performed by populating a predefined data abstraction sheet. This included the total number of patients and shoulders included in the study, number of female and male patients, average age of the patient cohort, hand dominance, os acromiale subtype (preacromion, mesoacromion, and meta-acromion), surgical procedure(s) performed, range of motion, patient-reported outcome scores, and complications. Several standardized outcome measurement tools were used across studies, including the Constant score, American Shoulder and Elbow Surgeons (ASES) Shoulder Score, University of California Los Angeles (UCLA) Shoulder Score, Penn Shoulder Score (PSS), and the Disabilities of the Arm Shoulder and Hand (DASH) Score.

### Statistical analysis

The summary statistics indicating the number of patients extracted from the individual studies was performed using counts, frequencies, and percentages where necessary. Data points are expressed as weighted means. A meta-analysis could not be performed due to the heterogeneity in data across studies, and as a result, no statistical tests were performed.

## Results

Nineteen studies meeting the inclusion criteria were identified and included in our final analysis [3–21]. Collectively, these studies account for 210 patients, with a total of 221 shoulders that underwent 221 surgeries for a symptomatic os acromiale. The average age of the included patients was 46 years (range, 19 to 78 years). Excluding 7 patients in the study performed by Edelson et al. [5], where patient gender was not reported, 65% of patients in this review were male. Of the 99 cases that reported hand dominance, 64% of surgeries were performed on the patient’s dominant arm. Of the 174 cases that reported the anatomical location of the os acromiale, 7 (4%) involved the preacromion, 164 (94%) the mesoacromion, and 3 (2%) the meta-acromion. With regard to concurrent surgical procedures performed at the time of the index surgery, rotator cuff repair was performed in 56% of cases (38% of patients within the excision group, 50% in the acromioplasty group, and 65% in the internal fixation group). In 9.5% of patients, a biceps tenodesis was performed in addition to the procedure used to address the symptomatic os acromiale. The mean postoperative follow-up was 32.2 months (range, 5 to 120 months). All surgical techniques used to manage the symptomatic os acromiale resulted in improved clinical outcomes, as measured using both subjective and objective methods. Details of each included study are presented in Table 1. All results are outlined in Tables 2 and 3.

**Table 1:** Characteristics of the included studies

Study	No. of patients	No. of shoulders^†^	Male:female	Mean age (range)	Dominant/non-dominant	Pre/Meso/Meta	Mean follow-up (months, range)	Surgical technique
Edelson et al. 1993 [5]	7	7	NR	NR	NR	NR	29 (18–40)	5 excision 2 ORIF (screws)
Hutchinson et al. 1993 [6]	3	3	1:2	24 (18–27)	NR	NR	15 (6–24)	1 excision 2 acromioplasty
Hertel et al. 1998 [17]	12	15	0	54 (37–63)	11:4	0/15/0	44 (13–72)	15 ORIF (K-wires)
Warner et al. 1998 [21]	14	15	7:7	57 (19–76)	NR	1/11/3	34 (24–47)	3 excision 3 ORIF (K-wires) 2 excision following failed ORIF (K-wires) 7 ORIF (screws)
Ryu et al. 1999 [18]	4	4	3:1	27 (20–43)	2:2	0/4/0	34 (12–84)	4 ORIF (screws)
Satterlee 1999 [20]	6	6	4:2	48 (29–63)	3:3	0/6/0	55 (36–72)	6 ORIF (screws)
Wright et al. 2000 [10]	12	13	8:4	36 (18–54)	NR	0/13/0	29 (20–72)	13 excision
Boehm et al. 2003 [3]	33	33	23:10	56 (44–70)	NR	3/30/0	41 (24–95)	6 excision 5 acromioplasty 22 ORIF (K-wires)
Abboud et al. 2006 [11]	19	19	12:7	53 (35–73)	13:6	0/19/0	40 (24–94)	11 acromioplasty 5 ORIF (K-wires) 3 ORIF (screws)
Pagnani et al. 2006 [9]	9	11	9:0	22 (18–25)	7:4	0/11/0	44 (24–78)	11 excision
Sahajpal et al. 2007 [19]	1	1	0:1	53	1:0	0/1/0	18	1 ORIF (screws)
Bedi et al. 2009 [15]	1	1	0:1	19	0:1	0/1/0	12	1 ORIF (screws)
Campbell et al. 2012 [4]	28	31	17:11	55 (21–78)	18:10	3/28/0	41 (9–85)	31 excision
Atoun et al. 2012 [13]	8	8	1:7	54 (38–67)	6:2	0/8/0	22 (12–36)	8 ORIF (absorbable screws)
Barbier et al. 2013 [14]	10	10	7:3	43 (16–65)	NR	0/10/0	48 (6–124)	10 ORIF (K-wires)
Johnston et al. 2013 [7]	6	6	4:2	53 (36–65)	3:3	0/6/0	25 (5–36)	6 excision
Kawaguchi et al. 2016 [8]	1	1	0:1	73	0:1	0/1/0	27	1 excision
Beliën et al. 2017 [16]	5	5	4:1	49 (20–67)	NR	NR	7.5 (5–13)	5 ORIF (plating)
Atinga et al. 2018 [12]	31	32	24:7	50 (21–74)	NR	NR	47 (12–120)	32 ORIF (screws)

*NR* not reported, *Pre* preacromion, *Meso* mesoacromion, *Meta* meta-acromion, *ORIF* open reduction and internal fixation

^†^Several patients underwent surgical management for symptomatic bilateral os acromiale

**Table 2:** Patient-reported outcome scores stratified by surgical category

Surgical technique	No. of patients	No. of shoulders	Patient-reported outcome scores					Subjective scores
			Score	No. of patients^†^	Preop*	Postop*	Change^ǂ^	Good or excellent (%)
Excision	71	77	ASES	31	43.3	92.1	48.8	
			Constant pain component	11	3.9	12.9	9.0	
			Constant total	11	NR	72.6	---	92% (59 of 64 responses)
			UCLA	14	16.8	31.3	14.5	
			PSS	6	50.6	78.5	27.9	
			QuickDASH	6	NR	15.9	---	
Acromioplasty	18	18	Constant pain component	18	4.5	12.5	8.0	63% (10 of 16 responses)
			Constant total	18	NR	73.9	---	
ORIF	121	126	ASES	6	38.8	93.1	54.3	
			Constant pain component	22	4.5	13.1	8.6	
			Constant total	45	52.5	76.1	23.6	
			UCLA	4	19	35	16	
			DASH	5	NR	37.3	---	82% (54 of 66 responses)
			QuickDASH	10	NR	20.6	---	

*ORIF* open reduction and internal fixation, *Preop* preoperative, *Postop* postoperative, *ASES* American Shoulder and Elbow Society, *UCLA* University of California Los Angeles, *DASH* Disabilities of Arm Shoulder and Hand, *PSS* Penn shoulder score, “---” change score not calculated

^†^Number of patients that completed patient-reported outcome scores within each category of surgical treatment

*The values are given as weighted means

^ǂ^Postoperative score minus preoperative score

**Table 3:** Active range of motion stratified by surgical category

Surgical technique	No. of patients	No. of shoulders	Range of motion (mean, range)					
			FE Preop	FE Postop	Change^†^	ER Preop	ER Postop	Change^†^
Excision	71	77	156.5 (143–170) (*n* = 7)*	166.5 (163–170) (*n* = 7)*	10	70 (70) (*n* = 1)*	70 (70) (*n* = 1)*	0
Acromioplasty	18	18	117 (102–132) (*n* = 11)*	145.3 (134–152) (*n* = 18)*	28.3	33.3 (22.5–44) (*n* = 11)*	42 (35–46) (*n* = 18)*	8.7
ORIF	121	126	125.7 (116–144.5) (*n* = 24)*	155.7 (141–165) (*n* = 24)*	30	53 (38–61) (*n* = 24)*	54.5 (37–64.5) (*n* = 24)*	1.5

The values are presented in degrees (weighted means, range)

*ORIF* open reduction and internal fixation, *FE* forward elevation, *ER* external rotation, *Preop* preoperative, *Postop* postoperative

*The number of patients with available data

^†^Postoperative score minus preoperative score

### Excision

Complete surgical excision of the os acromiale accounted for 35% of the included cases (77 of 221 cases) [3–10, 21]. A variety of objective outcome measures were used within the included studies, all of which demonstrated postoperative improvements (i.e., preoperative vs. postoperative scores). The mean ASES score improved from 43.3 to 92.1, Constant pain score from 3.9 to 12.9, UCLA score from 16.7 to 31.3, and Penn score from 50.6 to 78.5 (Table 2). Mean forward elevation improved by 10° while no change was observed in external rotation. Ninety-two percent of patients rated their overall subjective postoperative outcome as “good” or “excellent” (Tables 2 and 3).

### Acromioplasty

Acromioplasty accounted for only 8% of the included cases (18 of 221 cases) [3, 6, 11]. There was a mean improvement in the Constant pain score from 4.5 to 12.5 (i.e., preoperative vs. postoperative score) (Table 2). There were mean postoperative improvements in both forward elevation (28°) and external rotation (9°). Sixty-three percent of patients rated their overall subjective postoperative outcome as “good” or “excellent,” 25% as “satisfactory,” and 12% as “poor” (Tables 2 and 3).

### Open reduction and internal fixation

Open reduction and internal fixation was the most frequently performed surgery, accounting for 57% of the included cases (126 of 221 cases) [3, 5, 11–21]. Procedures performed within this category included internal fixation using Kirshner wires (*n* = 57) [3, 11, 14, 17], screws (*n* = 56) [5, 11, 12, 15, 18–21], plates (*n* = 5) [16], and absorbable screws (*n* = 8) [13]. A tension band construct was most commonly utilized (100 of 113 cases; 88%), whereby inter-fragmentary compression was obtained using two parallel Kirshner wires or cannulated screws combined with a cerclage or figure-of-eight wire [3, 11, 12, 14, 15, 17, 21] or suture [19, 20]. Sixty-seven patients in the fixation group (67 of 126 cases; 53%) received autogenous bone grafting, while 1 subject received HEALOS injectable bone graft replacement (DePuy, Raynham, MA, USA) [19], and 8 subjects received tri-calcium phosphate bone substitute [13]. Therefore, the majority (76 of 126 cases; 60%) of patients in the fixation cohort received concomitant autogenous bone graft or synthetic bone graft substitute.

When objective data was reported, there were improvements observed in active range of motion and patient-reported outcome scores (i.e., preoperative vs. postoperative scores) (Table 2). Specifically, the mean ASES score improved from 38.8 to 93.1, Constant pain score from 4.5 to 13.1, Constant total score 52.5 to 76.1, and UCLA score from 19 to 35 (Table 2). Mean improvements in active forward elevation and external rotation were 30° and 1.5°, respectively (Table 3). Eighty-two percent of patients rated their overall subjective postoperative outcome as “good” or “excellent.”

### Complications

A detailed description of complication type stratified by surgical category is presented in Table 4. There was a total of 87 reported complications among the three treatment categories studied. The excision group experienced a single surgical site infection that required reoperation (irrigation and debridement). The acromioplasty group experienced 2 deep surgical site infections requiring reoperation (irrigation and debridement). The ORIF group, in comparison, experienced a complication rate of 67% (84 of 126). Of the 84 reported complications in this treatment category, however, 34 (40.5%) were attributed to asymptomatic hardware removal. Of the 8 bio-absorbable screw fixation cases, there were 4 reported complications (1 nonunion, 2 symptomatic hardware, 1 iatrogenic fracture); of these patients, 2 required reoperation to trim the screws (i.e., decrease hardware prominence), 6 months after the first procedure. Patients that underwent plate fixation (*n* = 5) experienced 2 complications (i.e., symptomatic hardware), both requiring removal. There were 19 complications reported in those patients managed with cannulated screws including 1 nonunion, 1 surgical site infection requiring reoperation, 1 superficial wound dehiscence, 1 seroma formation, and 15 reoperations for hardware removal (Table 4). There was a total of 59 reported complications in patients managed with K-wires including 1 deep surgical site infection requiring reoperation, 2 superficial surgical site infections managed medically, 2 surgical failures (symptomatic nonunion) requiring revision surgery (i.e., fragment excision), 8 radiographic nonunions managed conservatively, 2 cases of complex regional pain syndrome, and 44 reoperations for hardware removal (Table 4).

**Table 4:** A detailed description of complication type stratified by surgical category

Complication	Excision (*n* = 77)	Acromioplasty (*n* = 18)	ORIF				
			K-wires (*n* = 57)	Cannulated screws (*n* = 56)	Plating (*n* = 5)	Absorbable screws (*n* = 8)	Total
Seroma				1			1
Wound dehiscence				1			1
Surgical site infection	1	2	3	1			7
Failure of index procedure			2				2
Nonunion			8	1		1	10
Iatrogenic fracture						1	1
Symptomatic hardware removal			20	5	2	2	29
Asymptomatic hardware removal			24	10			34
Complex regional pain syndrome			2				2
Total	1	2	59	19	2	4	87

*ORIF* open reduction and internal fixation

## Discussion

Operative management is often recommended for patients with a symptomatic os acromiale that have failed nonoperative treatment. A variety of surgical techniques are available; however, a universally accepted standard of treatment does not exist. In light of the current literature, best practice recommendations remain unclear and evidence-based recommendations are lacking.

Several surgical strategies have been described for the treatment of a symptomatic os acromiale. Excision has been described for both small (< 1.5 cm) and large (> 1.5) fragments, both with and without deltoid repair. Neer and colleagues [24] have reported generally poor outcomes and weakened deltoid performance following large acromion fragment excision given that it alters the fulcrum needed for deltoid function; careful repair of the deltoid attachment following os acromiale excision leads to improved patient outcomes [24, 25]. All nine studies included in this review, in which excision was undertaken, employed a deltoid-sparing or repair technique to ensure postoperative deltoid function would not be compromised. The success seen following acromioplasty (i.e., subtotal excision) is often attributed to the reduction of bony impingement secondary to the mobility of the os fragment. Determining the amount of mobility across the os fragment is difficult to quantify clinically, and as such, selecting patients who would benefit from an acromioplasty remains difficult. The use of ORIF has been previously described in the literature; greater union rates and fewer hardware removals can be seen with the use of cannulated screws compared to K-wire fixation.This is attributed to cannulated screws providing superior interfragmentary compression and a more rigid construct (vs. K-wires). Overall, ORIF techniques are performed in such a way that the acromion length and deltoid attachments are preserved, thereby maintaining deltoid strength and function.

All studies included in this review reported improved active range of motion following surgery. Forward elevation and external rotation improved across all treatment groups with the exception of external rotation in the excision group which remained unchanged. The most pronounced improvements in forward elevation were observed in both acromioplasty and ORIF treatment groups, while improvements in external rotation were greatest following acromioplasty.

Given the heterogeneity of standardized outcome scoring systems used across studies, it was difficult to directly compare the results between surgical groups. However, we can assess the results from each score individually using the previously published minimal clinically important difference (MCID) scores. Wylie et al. [26] report a MCID of 17 for the ASES score and 11.4 for the PSS when applied to rotator cuff pathologies and impingement. The excision and ORIF groups exceed the MCID for the ASES score, while both the acromioplasty and ORIF groups exceed the MCID for the PSS. The ORIF group demonstrated an improved postoperative Constant score of 23.5 points which is considered a clinically significant improvement [27]. Unfortunately, the UCLA score does not have published MCID data; however, we report improvements in postoperative scores using the UCLA system for both groups of patients who underwent excision and ORIF. Therefore, using these MCID scores, we can comfortably suggest that all three of the above surgical techniques lead to clinically relevant improvements in objective patient outcomes. Patient subjective outcomes across all included studies classified postoperative satisfaction into four categories of poor, fair, good, and excellent. The highest patient satisfaction rate was experienced by the excision group (92%), closely followed by the fixation group (82%). The acromioplasty group demonstrated good or excellent satisfaction in only 63% of patients; the remainder of patients reported fair or poor outcomes, which may be partly explained by a persistently painful nonunion.

When comparing complication rates, it is clear that ORIF carries the highest risk of complication with a reported 67% event rate. Of these, 40.5% were for asymptomatic hardware. Although potentially not considered a “true” postoperative complication, hardware removal does come with potential risks to the patient and adds significant cost to the health care system. Screw fixation accounted for 20% of symptomatic hardware requiring removal, while K-wire fixation accounted for the remainder. Within the ORIF group, the use of cannulated screws carries a lower overall complication rate (19 complications in 56 cannulated screw cases) whereby K-wire fixation carries the highest complication rate (59 complications in 57 K-wire cases). In addition, we report a 14% (8 of 57 cases) incidence of nonunion in patients treated with K-wire fixation compared to 2% (1 of 56 cases) in those treated with cannulated screws. Union was assessed radiographically at 6 months postoperatively. Nonunion is an important determinant of surgical outcome as it has been noted to be a primary cause of continued pain and patient dissatisfaction despite operative management [13].

This review is not without its limitations, specifically that it is fundamentally limited by the weaknesses of each included study. Sources of bias in this study include (1) small number of cases per study (and within treatment groups), (2) substantial heterogeneity in reported outcome scores, (3) disease-specific outcome scores do not exist for patients with symptomatic os acromiale, (4) performance bias resulting from the use of concomitant surgical interventions (i.e., rotator cuff repair, bone grafting) as well as technique variation across surgeons (most apparent within the ORIF group), and (5) short-term clinical follow-up. Furthermore, due to the retrospective nature of the included studies, the overall strength of the clinical recommendations was limited.

## Conclusions

This study reports on the largest sample of patients to date that have undergone surgical treatment for a symptomatic os acromiale. Excision of the os acromiale fragment with meticulous repair of the deltoid attachment leads to the greatest patient satisfaction while providing improvement in objective outcome measures and imparting the lowest risk of postoperative complications. This method of treatment may provide more consistent results in patients with small symptomatic os acromiale fragments (i.e., preacromial os); however, this review has demonstrated that excision of larger fragments (i.e., mesoacromial) can provide good to excellent surgical results. The role of acromioplasty (i.e., subtotal os excision) remains unclear, but is likely best reserved for patients with a stable symptomatic os and an associated rotator cuff tear that is addressed at the same time as the subacromial decompression. Similar to fragment excision, this method of treatment has a low complication risk. In patients with a large unstable fragment (i.e., mesoacromion) with limited or no rotator cuff disease, surgical fixation using cannulated screws may provide increased preservation of deltoid function while offering good to excellent patient satisfaction. However, patients must be informed that a second operation may be required to remove symptomatic hardware. Surgeons must also appreciate that hardware removal involves an additional risk to the patient and cost to the health care system.
